# STAR sleep recording export software for automatic export and anonymization of sleep studies

**DOI:** 10.1038/s41598-022-19892-0

**Published:** 2022-09-23

**Authors:** Sami Nikkonen, Henri Korkalainen, Juha Töyräs, Timo Leppänen

**Affiliations:** 1 Department of Applied Physics, University of Eastern Finland, Kuopio, Finland; 2 Diagnostic Imaging Center, Kuopio University Hospital, Kuopio, Finland; 3 School of Information Technology and Electrical Engineering, The University of Queensland, Brisbane, Australia

**Keywords:** Software, Data processing

## Abstract

Sleep research often relies on large retrospective clinical datasets. However, as the data is usually stored in proprietary formats specific for each sleep software, the raw data cannot be easily accessed and analyzed with external tools. While the raw data can usually be exported to more common data formats, this is often a cumbersome and labor-intensive task as it is not required for clinical purposes. Additionally, the recordings often include sensitive patient information which must be removed before the data can be shared or analyzed externally. This anonymization can be difficult to perform manually without the correct tools or knowledge of the file types and how they internally store the data. The STAR sleep recording export software provides a simple tool that can be used to perform the sleep study exports automatically. This allows the user to easily export a batch of sleep studies with minimal effort. In addition, the software can also be used to automatically anonymize the exported sleep recordings allowing researchers to save time and personnel resources as these do not need to be allocated for exporting and anonymizing sleep studies. The software supports Noxturnal, RemLogic, Profusion PSG and Sleepware G3 and it is free and openly available for anyone to download and use.

## Introduction

Poor sleep and sleep disorders are substantial global health problems with major social and economical burdens^1^. Sleep disorders are highly prevalent and for example, obstructive sleep apnea and insomnia alone are estimated to affect nearly half of the adult population to some degree^2,3^. Therefore, to alleviate the negative health effects of sleep disorders it is vital to enable efficient sleep research.

Sleep and sleep disorders are commonly investigated with overnight sleep studies. These sleep studies are multi-channel polysomnographic recordings that are usually manually scored using various sleep software. A sleep software connects to the acquisition system and records and stores the polysomnographic data. Usually, the software also allows scoring of sleep stages and respiratory events and generates reports based on the scorings. These reports usually form the basis of diagnosis and are mainly used for clinical purposes.

Sleep research commonly relies on retrospective clinical data. However, for research purposes, it is often necessary to have access to the raw data and the individual annotations so that they can be effectively and freely analyzed with more advanced external computational tools. However, as the clinical data is usually only stored in proprietary formats that are specific for each sleep software, the raw data is not directly accessible for data processing or signal analysis tasks.

Most sleep software commonly allow the data to be exported to general data formats such as European Data Format (EDF), Text (TXT), or Extensible Markup Language (XML) files. However, as the sleep software have mostly been developed for clinical purposes, this exportation is cumbersome and often cannot be done programmatically, or even in batches, and thus must be performed manually for each individual sleep study. It is common to have datasets with hundreds or even thousands of sleep studies making manual exportation a massive task and time-sink. To further complicate things, this export process is often buried deep into complex sub-menus with multiple settings, which increases the chance of mistakes caused by misclicks for example. Overall, these factors make manual sleep study exportation not only extremely time-consuming but also prone to consistency errors for large datasets.

It is often also necessary to remove all sensitive patient information from the exported sleep recordings so that they can be shared and analyzed with partners and collaborators. This can also be a complex and time-consuming task if performed manually and in some cases may be very difficult to perform without deep knowledge of the data file types and their internal structure. In addition, manual anonymization always carries a risk that some data is missed by accident and is still present in the recordings that are shared.

Therefore, the aim of this STAR sleep recording export (SSRE) research software is to provide an easy-to-use tool that will perform the sleep study exports automatically. The user can select a batch of sleep studies to export from a graphical user interface. The SSRE software will then cycle through each of the studies and automatically perform the necessary tasks to navigate the sleep recording software’s interface and perform the selected exports. In addition, the software can also optionally anonymize the exports automatically by removing all patient information from them. SSRE software thus allows researchers to easily export large datasets and utilize them in their research without the need to worry about export workload or the anonymization process.

## Methods

The SSRE software is mainly implemented in AutoHotkey (version 1.1.33.06)^4^. The anonymization of the exports is implemented with Matlab (version R2021b). The current version of the SSRE software supports Noxturnal (Nox Medical, Reykjavik, Iceland), RemLogic (Embla Systems, Kanata, Canada), Profusion PSG (Compumedics, Abbotsford, Australia) and Sleepware G3 (Philips Respironics, Murrysville, USA). The software was developed completely independently from the sleep recording software companies.

SSRE was developed and tested using Windows 10, Noxturnal 6.0.2, RemLogic 4.0.1, Profusion PSG V4.5 and Sleepware G3 3.9.7 with default settings and no user interface modifications. The software can be used with also other versions of the sleep recording software but as this has not been tested, the compatibility may vary. The SSRE software was developed for use in the Sleep Revolution project (EU Horizon 2020^5^). During the project, the software was extensively tested by exporting thousands of sleep recordings from several partner sites.

The SSRE software is hosted at Zenodo^6^. The software installation consists only of extracting the zip-archive to the desired folder. The archive contains the export program itself, data files needed to run the program, and the license terms of SSRE which must be followed when using the software. Installing and using the export portion of the software does not require admin privileges or the installation of any other software, apart from the sleep software associated with the recordings (RemLogic, Noxturnal, Profusion PSG or Slepware G3). However, the anonymization tool requires Matlab runtime R2021b, which can be downloaded and installed for free and without a Matlab license^7^.

## Software description

**Figure 1:**
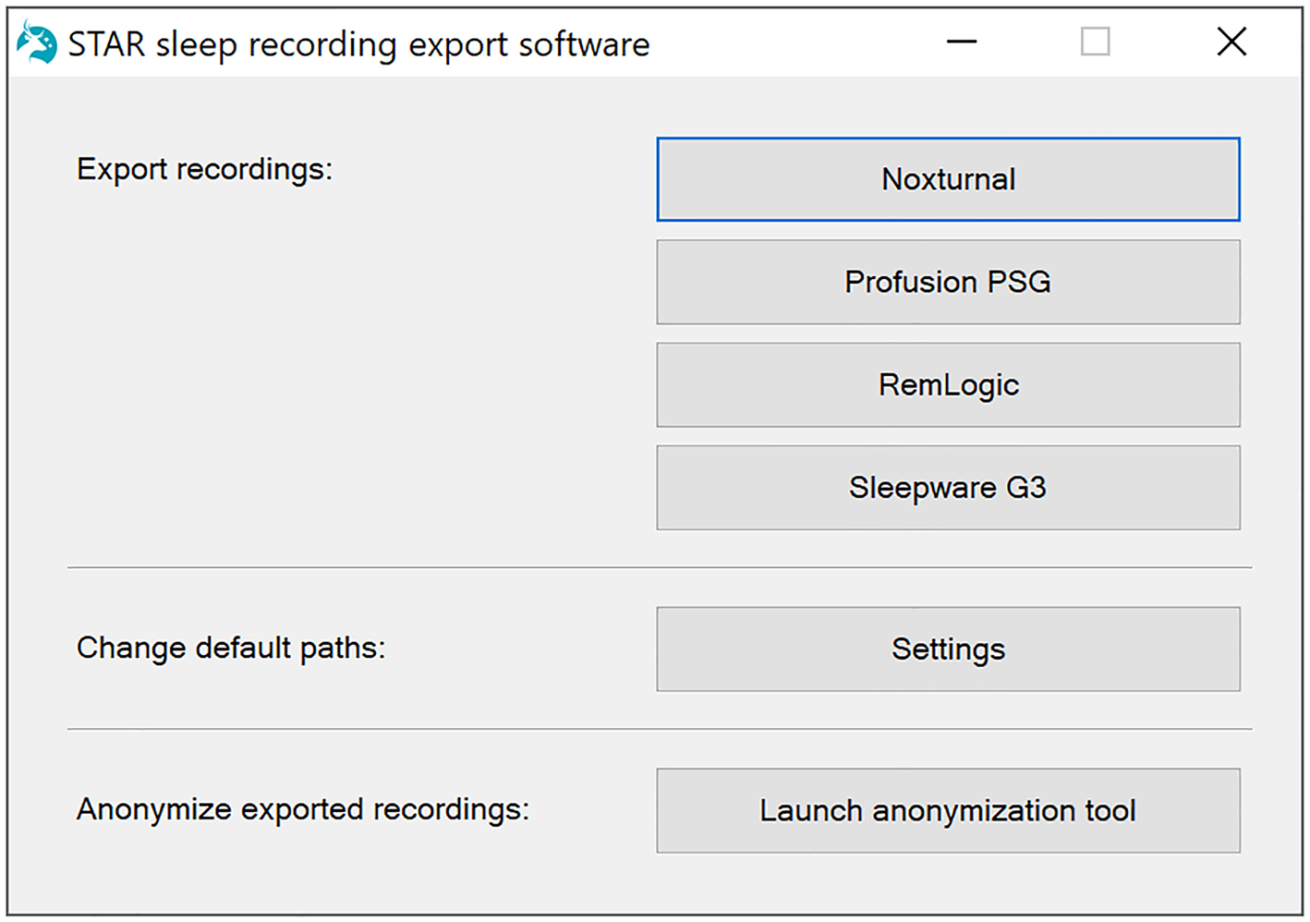
Main selection window.

The software is launched by running the ExportProgram.exe file. At first, the SSRE software shows the main selection window (Fig. 1). From here, the user can select which sleep software is appropriate for the recordings and should be used for the export. From the main selection window, the user can also launch the anonymization tool, which can be used to remove all patient information from the exported files. The anonymization process is described in more detail later. In addition, the user can change the default installation, recording, and export paths. These default paths can be used for more easily restarting the exports, but using them is completely optional.

**Figure 2:**
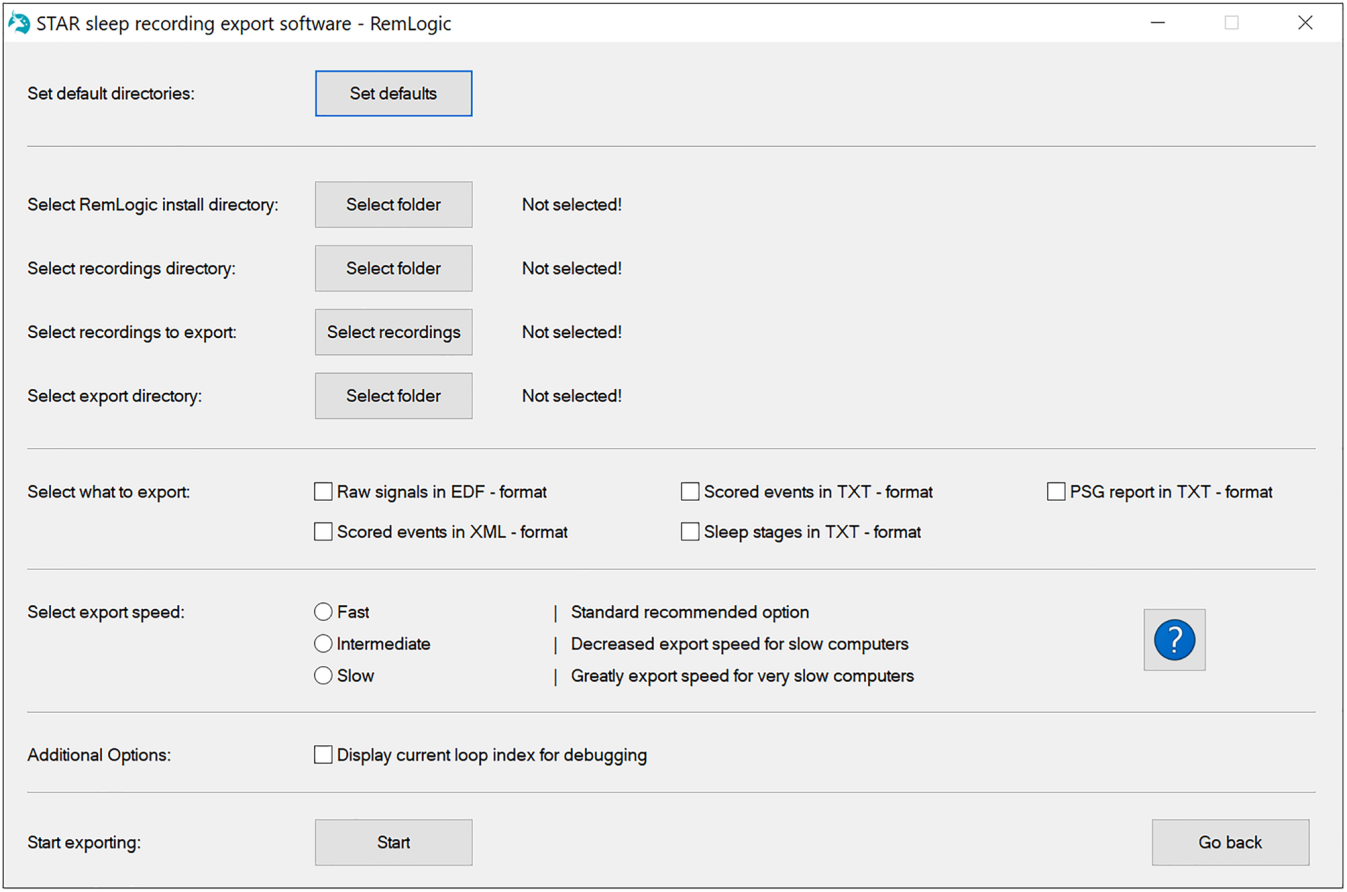
Export selection window for RemLogic recordings. Noxturnal, Profusion PSG and Sleepware G3 have the same main layout and functionality.

Selecting one of the available sleep software will bring up the export selection window (Fig. 2). From here, the user can select the installation path of the sleep software, the path of the folder containing the recordings, and the path where the user wishes the exports to be saved. Each of these paths can be selected from a “browse for folder” menu. Alternatively, the user can directly select default paths, which can be changed in the settings menu. The user can also go back to the main selection window by clicking “Go back”.

**Figure 3:**
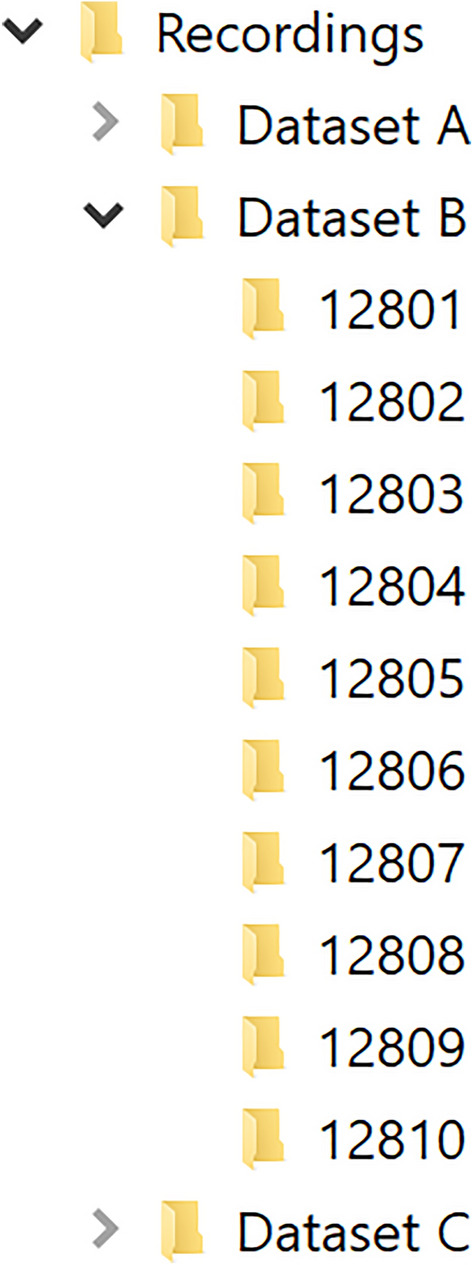
Correct folder structure for recordings. The recordings folder “Recordings/Dataset B” should contain all of the recordings to be exported. Each of the numbered subfolders (12801–12810) should contain the recording data for one measurement.

**Figure 4:**
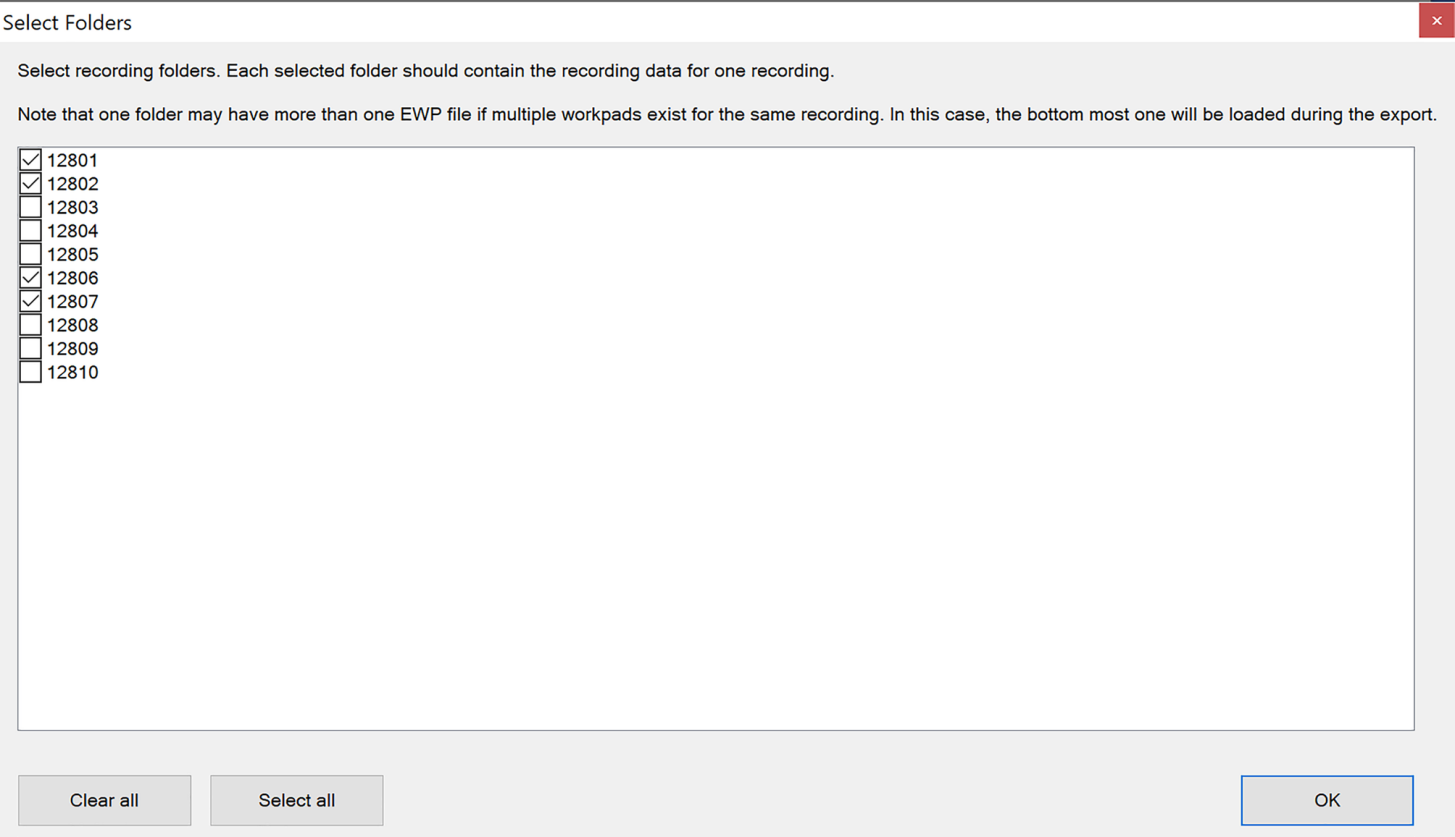
Recording selection window.

The recordings should be placed in a single folder so that each subfolder in this folder contains the recording data for one measurement. An example of a correct folder structure is presented in Fig. 3. The user can also select which individual recordings should be exported from the recordings directory. Figure 4 shows the interface for selecting the individual recordings. The user can either individually check which recordings should be exported, check all recordings, or uncheck all recordings. Clicking “OK” will bring the user back to the export selection window.

The user can also select what data will be exported. This will vary slightly depending on the export options allowed by the corresponding sleep software. In the RemLogic example case, the options are EDF signal export, TXT event export, PSG report, XML event export and TXT sleep stage export. The user can tick one or multiple of these boxes and the corresponding files will be generated during the export process.

Finally, the user can select a general “speed” parameter. A standard computer that is capable of running windows 10 and the sleep software in manual mode without issues can easily also handle the fastest export speed. However, some sleep centers may use dated hardware with limited capabilities for processing the sleep studies due to licensing or software support issues. In addition, the SSRE software may be used remotely where a bad connection might cause issues with the fastest speed. In these cases, slower export speeds can be selected for increased export time, but possibly higher compatibility. If a too fast speed is used on a slow computer, all of the simulated keystrokes may not be correctly registered, which might break the export as wrong options may be chosen. If the SSRE software detects an error during the export process, it will automatically stop execution and exit. The program will also automatically monitor the state of the export regardless of export speed and stop execution if an error is detected.

There are also a few additional options present only on specific sleep software. When exporting Noxturnal recordings, there is an option to automatically try to continue the export process if Noxturnal crashes which may occur when exporting very large datasets. If this option is selected, the SSRE software will attempt to automatically resume the export process when it detects Noxturnal crashing. The user can also select whether to attempt force closing the old Noxturnal instance when recovering from a crash. In most cases, this option can be left unchecked and the extra Noxturnal instances can be simply closed manually after the export is complete. However, if the computer running the exports has a low amount of RAM, it may be better to enable this option. When exporting Profusion PSG recordings, there is an additional option to perform an automatic desaturation scoring analysis before exporting the events. If this option is used, the user should be aware that this automatic scoring will not replace the old scoring so this option should only be used on data that has no previous desaturation scoring saved as this would result in duplicate desaturation events. There are also additional options for all sleep scoring software to display additional information during the export and generate an info file for each subject during the export. These options are intended for troubleshooting and can be ignored normally.

When all desired settings are chosen, the export can be started by clicking “Start”. A final pop-up will appear that reminds the user to not touch the keyboard or mouse during the export as this could break the export. However, the user can press “Esc” at any time to immediately abort the export and close the program. Clicking “OK” will open the corresponding export program and start exporting. Once the export is finished for all selected recordings, the SSRE software will inform the user and return to the export selection window.

After the exports have been completed, the anonymization tool can be used to remove all patient information from the exports. However, this anonymization step is completely optional and usually only required if the data will be shared. In addition, some exports may not require the anonymization process at all as the exports do not include patient information in the first place. It is also possible to also anonymize data that is not exported using the SSRE software as long as the exported data is in the same formats that the SSRE would also output. If the user wishes to perform the data anonymization, the anonymization tool can be launched from the main selection window.

**Figure 5:**
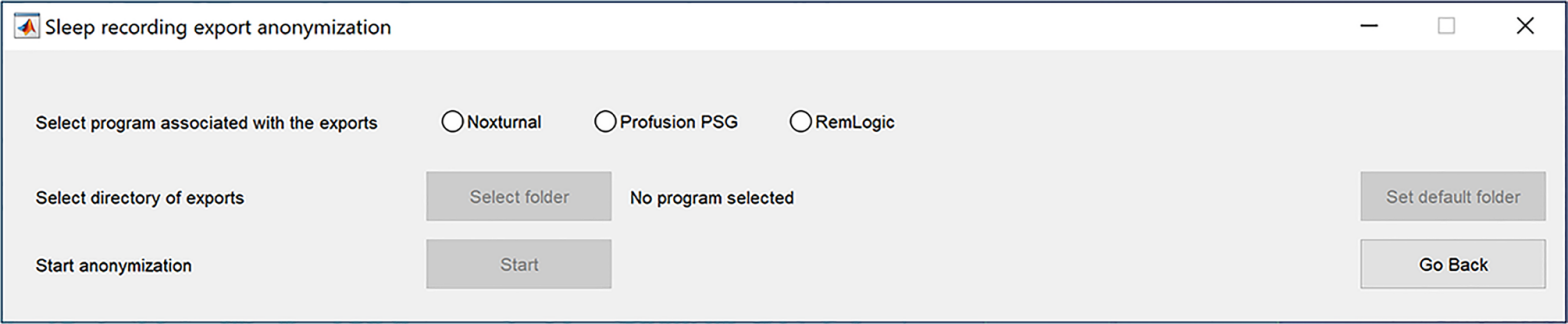
Anonymization selection window.

Once the tool launches, it opens the anonymization selection window (Fig. 5). From here, the user selects which program the exports were associated with, and which folder the exports are located in. If the default directories are used, the program will automatically use the same export folder as defined in the options. It should be noted that the program will perform the anonymization directly on the exports, and will not make another copy of them. Therefore, if the user wishes to back up the exports that still include the patient information, it should be done manually before the anonymization process. In addition, all export files in the folders will be anonymized. The program will automatically detect which files are present and sequentially anonymize them. Therefore, depending on which files were exported and the performance of the computer, the anonymization process can take from only a few ms/subject to as long as several minutes per subject.

**Figure 6:**
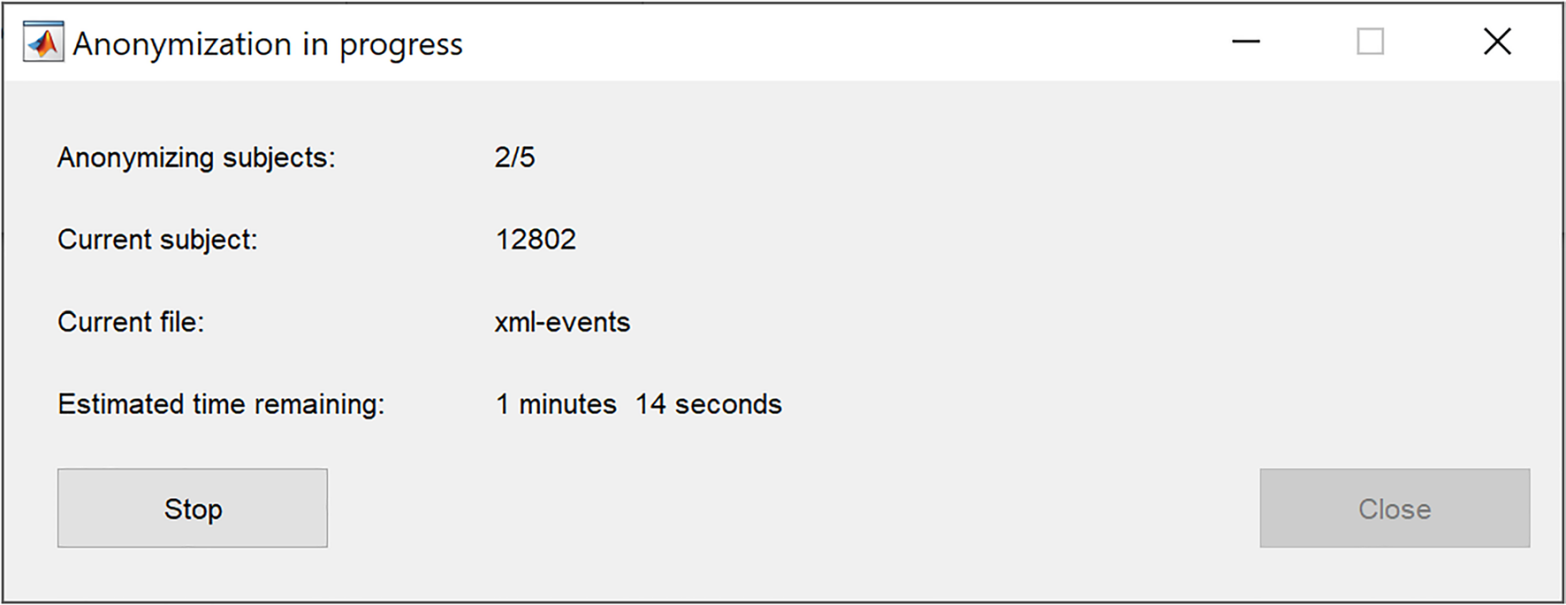
Anonymization progress.

Once the correct folder is selected, the user can press “Start” to begin the anonymization process, which will also open a progress window (Fig. 6). This window can be used to monitor the progress of the anonymization and the estimated time remaining. This window can also be used to prematurely stop the anonymization by pressing the “Stop” button. To ensure that the data is not corrupted by the stop-action, the anonymization of the current file will be finished and thus, the anonymization will not stop instantaneously. Therefore, the user should not attempt to force close the anonymization tool as this can lead to data corruption if the current write process is not allowed to complete. Once the “Close” button has been enabled, the window can be safely closed. When the anonymization is complete, the program will inform the user and at that point, the “Close” button will be again enabled and the program can be closed. It should be noted that the anonymization tool will not modify the names of the subject folders (12801–12810 in these examples). Therefore, the exports can still be linked to the original data and can only be considered pseudonymized.

## Impact

Although the SSRE software will not enable new types of research by itself, it simplifies and expedites the process of using retrospective clinical data for sleep research. Therefore, the SSRE software enables the use of new datasets that would be unfeasible to export manually due to a lack of personnel resources and time. In addition, if the data needs to also be anonymized, personnel with the necessary expertise to perform the anonymization, are required. In addition, since user input is constantly needed when performing the exports manually, it would be almost impossible to focus on other work at the same time. It is also very likely that some errors would be made during this process or some of the data could be accidentally exported with incorrect settings requiring additional troubleshooting and workload later. In comparison, if the export is performed with the SSRE software, the process requires very few personnel resources. The process will also take less time since the program does not need constant active supervision and can be running continuously even overnight and during weekends. Therefore, compared to a single person manually performing the exports full-time, the SSRE should also be around four times as fast (40-h work week vs. 168 total hours in a week).

In addition to internal testing, the SSRE software has already been deployed in the Sleep Revolution project^5^ to aid in exporting and anonymizing over 10 000 sleep recordings from 12 different sleep centers across Europe. Depending on used the hardware and the type of the recording, the data export process for a single subject typically takes around 3–10 min. Thus, if these 10 000 recordings would have been exported manually, the process would have taken over around 1000 working hours spent just on the exporting task. This would not only have added costs for the project but would not even have been feasible on this scale for most sleep centers due to them being short-staffed already. In addition, many centers may not have had the expertise required to anonymize the exported recordings as this is not a trivial task. Therefore, as data exportation and anonymization are required before the data transfer, the SSRE software has been highly beneficial for the project.

We expect that the SSRE software can be similarly helpful also in other research projects regardless of their size and enable data collection, sharing, and collaboration opportunities that might not have been possible otherwise. To the authors’ best knowledge, previous software with similar functions do not exist and have not been available previously.

## Conclusions

In conclusion, the SSRE is free and openly available software that can be used to easily export and anonymize data from sleep software to general data formats with minimal manual workload. The SSRE software currently supports Noxturnal, RemLogic, Profusion PSG and Sleepware G3. This automatic exporting will save time and resources while minimizing errors and inconsistencies that could be introduced with manual exporting.

## Data Availability

The SSRE software can be freely downloaded from Zenodo repository, https://zenodo.org/record/6139577.
